# Development of Nonaggregating Poly-A Tailed Immunostimulatory A/D Type CpG Oligodeoxynucleotides Applicable for Clinical Use

**DOI:** 10.1155/2015/316364

**Published:** 2015-08-25

**Authors:** Taiki Aoshi, Yasunari Haseda, Kouji Kobiyama, Hirotaka Narita, Hideaki Sato, Hirokazu Nankai, Shinichi Mochizuki, Kazuo Sakurai, Yuko Katakai, Yasuhiro Yasutomi, Etsushi Kuroda, Cevayir Coban, Ken J. Ishii

**Affiliations:** ^1^Laboratory of Adjuvant Innovation, National Institute of Biomedical Innovation, Health and Nutrition, Ibaraki, Osaka 567-0085, Japan; ^2^Laboratory of Vaccine Science, Immunology Frontier Research Center (iFReC), Osaka University, Suita, Osaka 565-0871, Japan; ^3^Vaccine Dynamics Project, BIKEN Innovative Vaccine Research Alliance Laboratories, Research Institute for Microbial Diseases (RIMD), Osaka University, Suita, Osaka 565-0871, Japan; ^4^Laboratory of Supramolecular Crystallography, Institute for Protein Research, Osaka University, Suita, Osaka 565-0871, Japan; ^5^GeneDesign Inc., Ibaraki, Osaka 567-0085, Japan; ^6^Department of Chemistry and Biochemistry, University of Kitakyushu, Kitakyushu, Fukuoka 808-0135, Japan; ^7^Corporation for Production and Research of Laboratory Primates, Tsukuba, Ibaraki 305-0843, Japan; ^8^Tsukuba Primate Research Center, National Institute of Biomedical Innovation, Tsukuba, Ibaraki 305-0843, Japan; ^9^Laboratory of Malaria Immunology, Immunology Frontier Research Center (iFReC), Osaka University, Suita, Osaka 565-0871, Japan

## Abstract

Immunostimulatory CpG ODNs have been developed and utilized as TLR9-dependent innate immune activators and vaccine adjuvants. Four different types of immunostimulatory CpG ODNs (A/D, B/K, C, and P type) have been reported. A/D type ODNs are characterized by high IFN-*α* production but intrinsically form aggregates, hindering its good manufacturing practice grade preparation. In this study, we developed several D35-derived ODNs (a commonly used A/D type ODN), which were modified with the addition of a phosphorothioate polynucleotide tail (such as dAs40), and examined their physical properties, solubility in saline, immunostimulatory activity on human PBMCs, and vaccine adjuvant potential in monkeys. We found that two modified ODNs including D35-dAs40 and D35core-dAs40 were immunostimulatory, similar to original D35 in human PBMCs, resulting in high IFN-*α* secretion in a dose-dependent manner. Physical property analysis by dynamic light scattering revealed that both D35-dAs40 and D35core-dAs40 did not form aggregates in saline, which is currently impossible for the original D35. Furthermore, D35-dAs40 and D35core-dAs40 worked as better vaccine adjuvant in monkeys. These results suggested that D35-dAs40 and D35core-dAs40 are two promising prototypes of nonaggregating A/D type ODN with advantages of ease of drug preparation for clinical applications as vaccine adjuvants or IFN-*α* inducing immunomodifiers.

## 1. Introduction

Immunostimulatory CpG oligodeoxynucleotides (ODNs) have been developed and utilized as Toll-like receptor (TLR) 9-dependent innate immune activators and vaccine adjuvants for more than 10 years [1]. Based on their backbone and sequence characteristics, immunostimulatory ODNs can be divided into four proposed different types (or classes) [2, 3]: A/D, B/K, C, and P-type ODNs. A/D types ODNs (mostly phosphodiester backbone with poly G tail at 3′ end) mainly stimulate interferon- (IFN-) *α* production from plasmacytoid dendritic cells (pDCs). B/K type ODNs (all phosphorothioate backbone) activate B cells and interleukin- (IL-) 6 production, and C type ODNs can stimulate both IFN-*α* and IL-6 despite IFN-*α* induction being lower than that by A/D type ODNs. Recently another P-type ODN has been described [4]. P-type ODN (all phosphorothioate backbone) contains two palindromic sequences with a cytokine profile similar to C type ODNs but with higher IFN-*α* production [4].

Although many different immunostimulatory ODNs have been developed, the most characteristic difference among them is their IFN-*α* induction profile. In this sense, A/D (high IFN-*α* profile) type and B/K (low IFN-*α*, high IL-6 profile) type ODNs are considered as two distinct and typical prototypes of immunostimulatory ODNs [5]. A recent microarray study also supported overlapping but different gene signatures between A/D and B/K type ODNs [6]. A/D type ODNs were dominantly characterized by prolonged induction of type I IFNs while B/K type ODNs induced many genes that were significantly associated with resistance to bacterial infection such as IL-1*β* and IL-6 [6]. These* in vitro* profile differences might reflect* in vivo* observed differences when these ODNs are utilized as a vaccine adjuvant or a monoimmunotherapeutic drug. In the case of a malaria vaccine, K3 (a B/K type ODN) showed better adjuvanticity for antibody production than D35 (an A/D type ODN), when it was added to the SE36/AHG immunization in cynomolgus monkeys [7]. However, A/D type ODNs induced better protective immune responses with heat-killed* Leishmania*/AHG vaccine compared to B/K type ODNs in rhesus monkeys [8]. Similarly, as a monotherapeutic drug for Leishmaniasis, A/D type ODNs also showed a better potential in both healthy and SIV-infected rhesus monkey models than B/K type ODNs [9]. Interestingly, in this model, B/K type ODN administration promoted the pathology of cutaneous Leishmaniasis [9].

Strong IFN-*α* induction with A/D type ODNs has been closely linked to the higher order structures of this type of ODN. The poly-G tail of A/D type ODNs forms G-quadruplex DNA structures in a salt solution, resulting in nanoparticle/aggregate formations [10–13]. Similarly, IFN-*α* inducing P-type ODNs formed dimeric structures or aggregates [4]. It has been repeatedly reported that aggregation formation is necessary for high IFN-*α* production with A/D type ODNs [12, 13], and this greatly hampers the clinical application of A/D type ODNs because autonomous development of ODN multimerization leads to uncontrolled aggregation and precipitation, resulting in high product-by-product differences and administration difficulties. To overcome such problems, introduction of thermolytic protective groups in A/D type ODNs has been attempted [14, 15]. This modification prevented aggregate formation in saline before administration, but, after* in vivo *administration, temperature-dependent cleavage of the protective groups allows G-quadruplex formation [14, 15]. This thermolytic pro-D type ODN strategy is a promising method for clinically applicable A/D type ODNs, but its feasibility for clinical application needs to be evaluated. Currently, no human clinical trials have been reported.

In this study, we developed nonaggregating A/D type ODNs by modifying original D35 and examined their physical and biological characteristics. We found that a simple modification of D35, such as the addition of a phosphorothioate polydeoxynucleotide at the 3′ end, strongly prevented aggregate formation in saline but maintained its high IFN-*α* inducing property.

## 2. Materials and Methods

### 2.1. Human PBMC Preparation

PBMCs were prepared from Japanese healthy adult volunteers with informed consent. All experiments using human PBMCs were approved by the Institutional Review Board of the National Institute of Biomedical Innovation (Permit number: 44). After preparation of PBMCs using Ficoll-Paque PLUS (GE) and LeucoSep (Greiner), they were plated at a concentration of 2 × 10^7^ cells/mL (96-well flat plates, in a total volume of 100 *μ*L/well) in RPMI 1640 medium supplemented with 10% fetal calf serum, 100 unit/mL penicillin, and 100 *μ*g/mL streptomycin (all from Nacalai, Japan).

### 2.2. CpG ODNs Stimulation with or without N-[1-(2,3-Dioleoyloxy)propyl]-N,N,N-trimethylammonium Methylsulfate (DOTAP)

The ODNs listed in Table 1 were synthesized by GeneDesign (Osaka, Japan). PBMCs were stimulated with the indicated concentrations of K3, D35, D35-dAs40, and other ODNs (Table 1) for 24 h. Stimulation by ODNs with DOTAP (Roche) was performed according to the manufacturers' instructions. Briefly, ODNs solution in serum-free medium (Opti-MEM; Gibco) and DOTAP solution in Opti-MEM were separately prepared and maintained for 15 minutes at room temperature before ODNs and DOTAP solutions were thoroughly mixed by pipetting. The resultant ODNs/DOTAP mixture was maintained for 15 minutes at room temperature. ODNs/DOTAP mixtures (100 *μ*L) were added to human PBMCs (2 × 10^6^ cells/100 *μ*L/well). 24 hours later, the supernatants were assayed for presence of cytokines.

### 2.3. Measurement of Cytokines by Enzyme-Linked Immunosorbent Assay

Cytokines in supernatants were measured using a pan-IFN-*α* enzyme-linked immunosorbent assay (ELISA) kit (Mabtech or PBL) and human IL-6 ELISA kit (DuoSet; R&D Systems) according to the manufacturers' instructions. ELISAs were developed with 3,3′,5,5′-tetramethylbenzidine (TMB; KPL). In some experiments, Milliplex assay (MPXHCYTO60KPMX26; Millipore) was also performed according to the manufacturer's instruction.

### 2.4. Dynamic Light Scattering (DLS)

DLS measurements were performed with a Wyatt DynaPro PlateReader II (Wyatt Technology, USA). Samples (1 mg/mL in PBS, stored for more than 18 hours) were measured (20 acquisitions at 25°C) in a 384-well plate (20 *μ*L/well). The polydispersity, hydrodynamic radius, and molecular weight were analyzed by Dynamics software v7.1.7.16 (Wyatt Technology, USA).

### 2.5. Transmission Electron Microscopy (TEM)

D35 (1 mg/mL in PBS) was dropped (10 *μ*L) on a Formvar-carbon-coated grid and incubated for 2 hours for adhesion to the grid. For negative staining, samples were washed with distilled water three times, and then a drop of 2% (wt/vol) uranyl acetate (pH 4.0) was placed on the grid and left to air dry. The grids were examined at a magnification of ×10,000 by an electron microscope (Hitachi H-7650).

### 2.6. Complexation of CpG ODN and Schizophyllan (SPG)

Alkaline denatured schizophyllan (SPG) (Mw. 150,000) solution (15 mg/mL in 0.25N NaOH) was added to the ODN solution (100 *μ*M in NaH_2_PO_4_) and then mixed thoroughly. The mixture was kept at 4°C overnight to complete the complexation. The molar ratio (SPG : DNA) was fixed at 0.27. Complexation efficiency between ODN and SPG was estimated from the residual-free ODNs in the mixed solution by using a MultiNA microchip electrophoresis system (Shimadzu, Japan).

### 2.7. Cynomolgus Monkey Immunization

Cynomolgus monkeys (*Macaca fascicularis*) were obtained and maintained at Tsukuba Primate Research Center in the National Institute of Biomedical Innovation (NIBIO). All experiments were performed under the protocol approved by the Committee on the Ethics of Animal Experiments of NIBIO (Permit number: DS22-4R1), and all efforts were made to minimize suffering. Cynomolgus monkeys (weight: 2-3 kg) were subcutaneously (s.c.) administered influenza split vaccine (SV) (5 *μ*g) (A/New Caledonia/20/99, BIKEN), with or without K3 (4.7 nmol = 30 *μ*g), D35 (4.7 nmol = 30 *μ*g), D35-dAs40 (4.7 nmol = 92 *μ*g), D35core-dAs40 (4.7 nmol = 80 *μ*g), or D35-SPG (4.7 nmol as D35-dAs40 amount) in a volume of 500 *μ*L of saline at days 0 and 14. Sera were collected at 4 and 8 weeks after the first immunization. Anti-SV total IgG in the serum was measured by ELISA. Each serum sample was serially diluted and the antibody titer was calculated as a reciprocal number of the dilution at 0.2 of OD450.

### 2.8. Statistical Analysis

Statistically significant differences were calculated using Graphpad Prism 5 software. A paired *t*-test was used for cytokine analysis and a two-tail nonparametric Mann-Whitney *U* test was for antibody titer analysis.

## 3. Results

### 3.1. Poly-dAs40-Tailed A/D Type ODNs Have Similar Immunostimulatory Potential to Original D35

**Figure 1:**
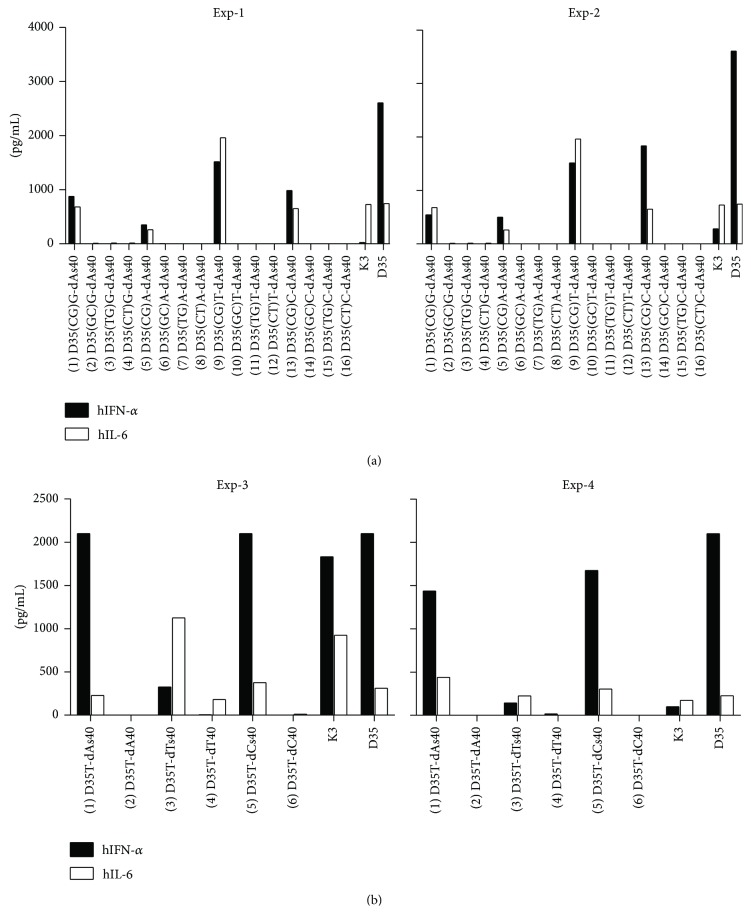
Screening of human PBMC with several different polynucleotide tailed A/D type ODNs. Human PBMCs were stimulated with the indicated synthetic ODNs (1 *μ*M) (Table 1) for 24 hours. IFN-*α* and IL-6 production in supernatants was measured by ELISA. (a) The requirement of CpG-motifs and G-hexamers for cytokine secretion from human PBMCs. Both IFN-*α* and IL-6 were secreted in a CpG motif dependent but G-hexamer sequence independent manner. (b) Comparison of tail backbones (phosphorothioate or phosphodiester). Cytokine secretion was dependent on phosphorothioate 40-mer tails (1, 3, and 5). ODNs with phosphodiester 40-mer tails showed almost no biological activity (2, 4, 6). The bar graphs indicate the concentrations from a single well of each stimulation. The results are representative of three different experiments consisting of two different donors (Exp-1 and Exp-2, Exp-3 and Exp-4).

We previously developed K3-SPG, a second generation B/K type CpG adjuvant, which is a particulate soluble complex of K3 CpG-ODN and schizophyllan (SPG) [16, 17]. To form complexes between K3-ODN and SPG, K3 ODNs have to be modified by adding a phosphorothioate 40-mer of deoxyadenylic acids (dAs40 tail) at the 3′ ends where SPG and dAs40 tails form triple-helix complexes [16]. In parallel with these experiments, we have also synthesized similarly tailed D35 ODNs, and tested their immunostimulatory activities (IFN-*α* and IL-6 induction) on human PBMCs. Cytokine ELISAs revealed that D35 (A/D type CpG-ODN) with an additional dAs40 tail at the 3′ end was similarly active as the original D35 (Figure 1(a); sample 1 versus D35). Each ODN sequence used in this study is shown in Table 1. Subsequent experiments further revealed that some D35-derived ODNs with a dAs40 tail were also biologically active, even with replacement of the 3′ G-hexamer to A-, T-, and C-hexamer (Figure 1(a); samples 5, 9, and 13). This is relatively unexpected, because the G-hexamer formed quadruplex structures via Hoogsteen base pairing, resulting in self-aggregates [10, 11], and it is believed that G-hexamer-mediated aggregation is an obligatory requirement for the biological activity of A/D type CpG ODNs [5, 12–14]. On the other hand, the immunostimulatory activities of D35-derived ODNs on human PBMCs are totally dependent on the presence of the CpG sequence. Replacement of CpG motif to GC, TG, and CT completely suppressed the ODNs' immunostimulatory activities (Figure 1(a); sample 9 versus samples 10–12), consistent with the unmethylated CpG-motif theory [18]. These results indicated that the presence of the G-hexamer sequence is not a necessary requirement for A/D type ODN immunostimulatory activities, whereas the activities of D35-derived ODNs was dominantly dependent on the presence of the CpG motif sequence.

### 3.2. Phosphorothioate Polynucleotide Tail Is Required for D35-Derived ODN Immunostimulatory Activity

The addition of a G-hexamer in phosphodiester CpG ODNs has been shown to increase ODNs' stimulatory activities by improving their cellular uptake [19, 20]. It has also been known that phosphorothioate ODNs are bound to proteins nonspecifically, and phosphorothioate CpG ODNs are more efficiently taken up by cells than phosphodiester CpG ODNs [21]. These reports suggested that the addition of dAs40 tails to D35-derived ODNs may improve their cellular uptake by nonspecific binding feature of the dAs40 tail's phosphorothioate backbone. Based on this hypothesis, we examined the requirement of the chemical backbone structure, by comparing the immunostimulatory activities of D35T-dAs40 and D35T-dA40 (original G-hexamer replaced with T-hexamer, and the tails were composed of either phosphorothioate or phosphodiester poly-A40) on human PBMCs (Figure 1(b); sample 1 versus sample 2). The biological activities of the ODNs were strongly dependent on the presence of the phosphorothioate tail, and almost no cytokine responses were observed by addition of phosphodiester tailed ODNs (Figure 1(b); sample 1 versus sample 2). We also tested other polynucleotide 40-mer tails such as poly-T(s)40 or poly-C(s)40 instead of poly-A(s)40 and found that the cytokine responses were generally independent of the bases of the polynucleotide tails but were dependent on the presence of the phosphorothioate backbone (Figure 1(b); samples 3–6), although relatively stronger cytokine responses were seen for addition of dAs40 and dCs40 compared to dTs40 tails (Figure 1(b); samples 1, 3, and 5).

### 3.3. DOTAP Compensates for the Absence of a G-Hexamer Sequence

**Figure 2:**
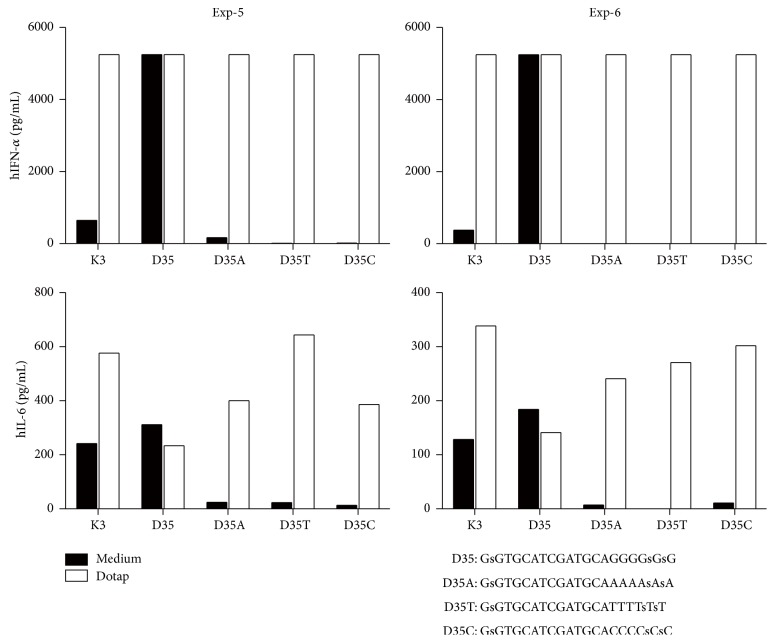
G-hexamer-less D35 ODNs required DOTAP for their immunostimulatory activities. Human PBMCs were stimulated with the indicated synthetic ODNs (1 *μ*M) with or without DOTPA for 24 hours. IFN-*α* and IL-6 productions in the supernatants were examined by ELISA. DOTAP revived the immunostimulatory activities of D35A, D35T, and D35C; those are A/D type ODNs which do not contain G-hexamer. These ODNs did not show any immunostimulatory activities without DOTAP. The bar graphs indicate the concentrations from a single well of each stimulation. IFN-*α* production overed the ELISA measurement maximum (ca. 5000 pg/mL) with DOTAP in Exp5 and Exp6.

We also tested the immunostimulatory activities of A-, T-, and C-hexamers containing D35 without phosphorothioate polynucleotide tails on human PBMCs (Figure 2). Consistent with previous reports [5, 22, 23], the immunostimulatory activity of original D35 was dependent on the presence of a G-hexamer (Figure 2). A-, T-, and C-hexamer-containing D35 (D35A, D35T, and D35C) had no effect on human PBMCs (Figure 2; black bar). In contrast, the same ODNs became comparatively active when they were added with DOTAP (Figure 2; white bar), a cationic lipid (N-[1-(2,3-dioleoyloxy)propyl]-N,N,N-trimethylammonium methylsulfate) that has been used for targeting CpG-ODNs to certain endosome compartments where TLR9-mediated signaling starts [24, 25]. This result indicated that the biological activity of A/D type ODNs does not require a G-hexamer sequence if ODNs are targeted to cellular uptake and appropriate intracellular compartments by DOTAP.

Taken together, these results suggested that the immunostimulatory activities of A/D type CpG-ODNs could be regulated by two separate processes: first, efficient cellular uptake through either G-hexamer aggregates or nonspecific binding via phosphorothioate polynucleotide tails and, second, the presence of a CpG motif that induces TLR9-dependent signaling. The fact that DOTAP can convert nonactive G-hexamer-less D35 such as D35A, D35T, and D35C into an immunostimulatory active compound suggests that the presence of a G-hexamer sequence itself is not necessary for CpG motif/TLR9 molecular interactions.

### 3.4. dAs40-Tailed D35 ODNs Do Not Form Large Aggregates

**Figure 3:**
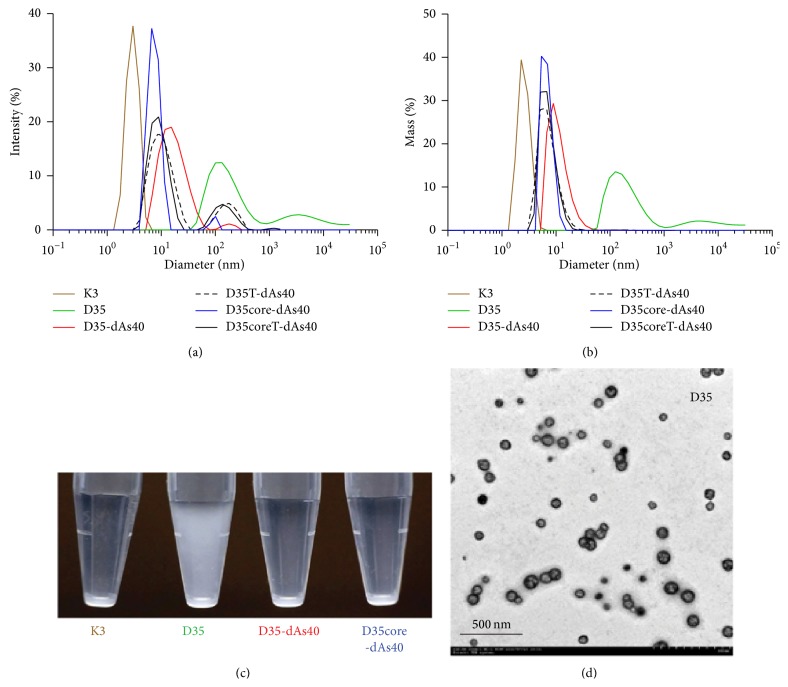
Physical properties of polynucleotide tailed A/D type ODNs in PBS solution. DLS analysis of the indicated ODN solutions (each 1.0 mg/mL in PBS) by % intensity plot (a) or % mass plot (b). See Table 2 for each measurement. (c) Turbidity status of the indicated ODN solution. The indicated ODNs were initially dissolved in distilled water at a concentration of 10 mg/mL (all ODNs were completely solubilized with water and the solutions were clear) and further diluted with PBS at a final concentration of 1.0 mg/mL. Solutions were stored at 4°C for at least 18 hours and then images were captured. D35 developed visible white turbidity during this incubation. In contrast, other ODNs were clear. (d) TEM image of aggregated D35 in PBS similarly prepared as in (c).

We also examined several dAs40-tailed D35-related ODNs for their physical properties with DLS (Figure 3). Good manufacturing practice (GMP) grade synthesis of clinically applicable A/D type ODNs has been hampered by G-tail dependent multimerization that results in uncontrollable polymorphisms, aggregation, and precipitation of ODN products [14] (Figures 3 and 8). Original D35 (1 mg/mL) showed variable and heterogenous aggregate formations in PBS resulting in visible turbidity within 24 hours (Figure 3(c)). Of note, this turbidity was not observed in D35 in water. DLS analysis revealed that this turbidity consisted of broadly distributed aggregates (size range from around 50 nm to more than 1 *μ*m in mean diameter; Figures 3(a) and 3(b)). In contrast, the same concentration of D35-dAs40 and D35core-dAs40 in PBS did not form visible aggregations (Figure 3(c)), and the size of the ODNs was less than 20 nm with a sharp peak in DLS (Figures 3(a) and 3(b) and Table 2). TEM analysis confirmed the DLS results and indicated that many globular particle sizes around 50–200 nm were distributed separately or formed stringed clusters of several particles (Figure 3(d)), consistent with a previous report [10, 11]. These pieces of data indicated that the addition of dAs40 tail greatly improved the physical uniformity of A/D type ODNs in PBS, even those containing a G-hexamer sequence such as D35-dAs40 showed virtually no aggregation with the dAs40 tail. We did not observe any meaningful structures in K3, D35-dAs40, D35T-dAs40, D35core-dAs40, and D35coreT-dAs40 by TEM.

### 3.5. dAs40-Tailed D35-Related ODNs Induce IFN-*α* in a Dose-Proportional Manner

**Figure 4:**
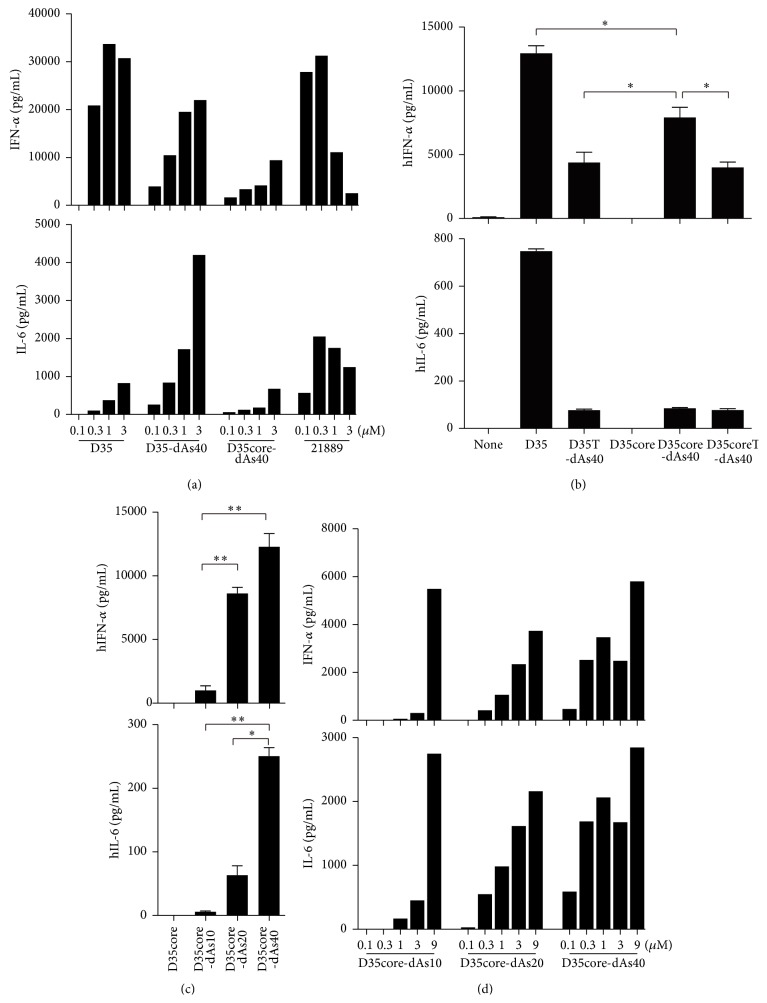
Biological activities of D35-dAs40 and D35core-dAs40. (a) Dose-dependent IFN-*α* production from human PBMC by the indicated ODNs. (b) Effect of the adjacent sequence of cytokine inducing activity. D35core plus dAs40 is sufficient to induce IFN-*α* secretion from human PBMC. Bar graph indicates mean ± SEM in triplicate. (c) dAs tail length affects the biological activity of D35core-dAs type ODNs. Human PBMC stimulated with the indicated ODNs (final concentration = 1 *μ*M), and after 24 h cytokine concentration was determined by ELISA. Bar graph indicates mean ± SEM in triplicate. (d) The tail length and ODN dose relations of the indicated ODNs. The bar graph shows dose-dependent IFN-*α* and IL-6 production from a single well by each stimulation. ^∗^ *P* < 0.05; ^∗∗^ *P* < 0.01.

We further evaluated the immunostimulatory activities of these ODNs by dose titration (Figure 4(a)). All ODNs including D35, D35-dAs40 (containing G-hexamer sequence), and D35core-dAs40 (without G-hexamer sequence) induced increased IFN-*α* and IL-6 responses from human PBMC in a dose-dependent manner (Figure 4(a)). In contrast, a recently reported P-type ODN, 21889, consisting of a phosphorothioate backbone containing two tandem palindromic sequences that promote the formation of dimeric structure or aggregates [4] showed decreased IFN-*α* and IL-6 responses when higher concentrations were used (Figure 4(a)).

### 3.6. D35core Plus dAs40-Tail Is Sufficient for IFN-*α* Production

We also examined the effect of the adjacent sequence from the D35core 12-mer, such as the 5′GG-sequence and 3′T-hexamer on IFN-*α* production (Figure 4(b)). The core sequence of D35 (12-mer) alone did not induce cytokine responses (Figure 4(b)) whereas the addition of a dAs40 tail to the 12-mer was sufficient to induce comparable amounts of IFN-*α*. Importantly this result was obtained in the absence of DOTAP. The presence of a T-hexamer seemed to decrease the biological activity. These results suggested that the core adjacent sequences such as the 5′GG-sequence and 3′T-hexamer were not necessary for this type of ODN immunostimulatory activities (Figure 4(b)).

### 3.7. Longer Phosphorothioated-A-Tailed ODNs Have Increased Immunostimulatory Activity

We also examined the effect of the length of the phosphorothioate A-polymer tail on immunostimulatory activity. When human PBMCs were stimulated with the same amount of the indicated ODNs (1 *μ*M), IFN-*α* and IL-6 production was positively correlated with the length of the dAs-tail (Figure 4(c)), suggesting that longer-tailed ODN could be more efficiently taken up. When the effect of each ODN concentration was examined, the increased concentration of each ODN (maximum tested 9 *μ*M) ultimately reached similar levels of IFN-*α* and IL-6 production (Figure 4(d)). Taken together, these data suggested that addition of phosphorothioate polynucleotide tails into a CpG motif containing short ODNs is a useful strategy to produce GMP applicable A/D type ODNs with controlled physical properties.

### 3.8. More Detailed Requirements and Characterizations of the Phosphorothioate Polynucleotide Tails

**Figure 5:**
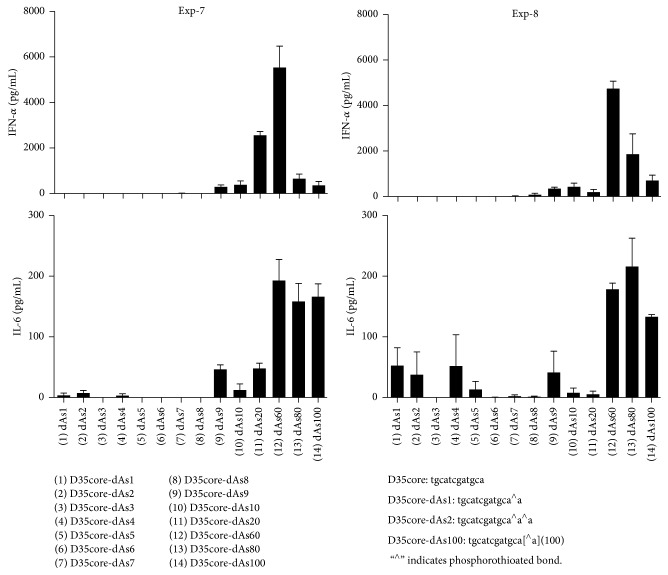
Length of the tail affects ODN's immunostimulatory activities. Human PBMCs were stimulated with the indicated synthetic ODNs (1 *μ*M) for 24 hours. IFN-*α* and IL-6 production in the supernatants were examined by ELISA.

**Figure 6:**
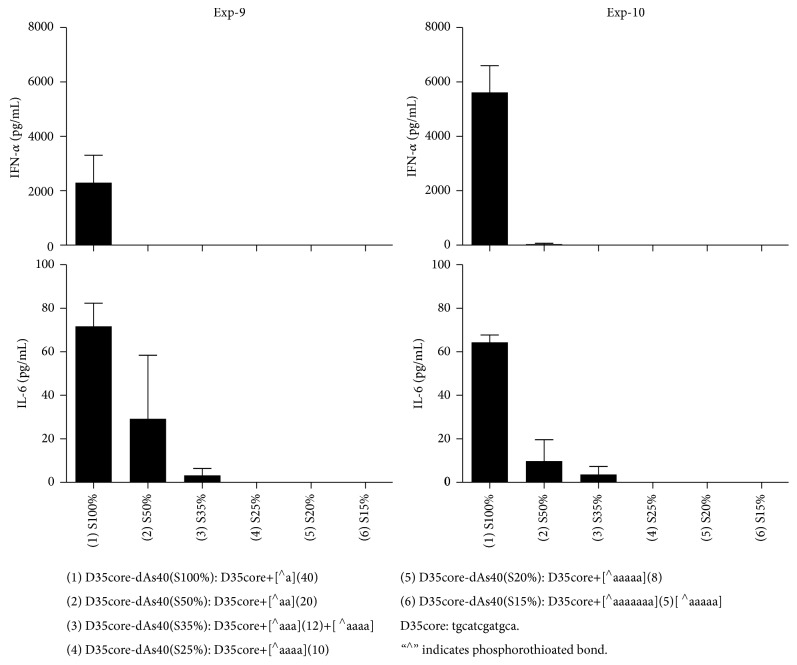
Phosphorothioation amount of the tail affects ODN's immunostimulatory activities. Human PBMCs were stimulated with the indicated synthetic ODNs (1 *μ*M) for 24 hours. IFN-*α* and IL-6 production in the supernatants were examined by ELISA.

**Figure 7:**
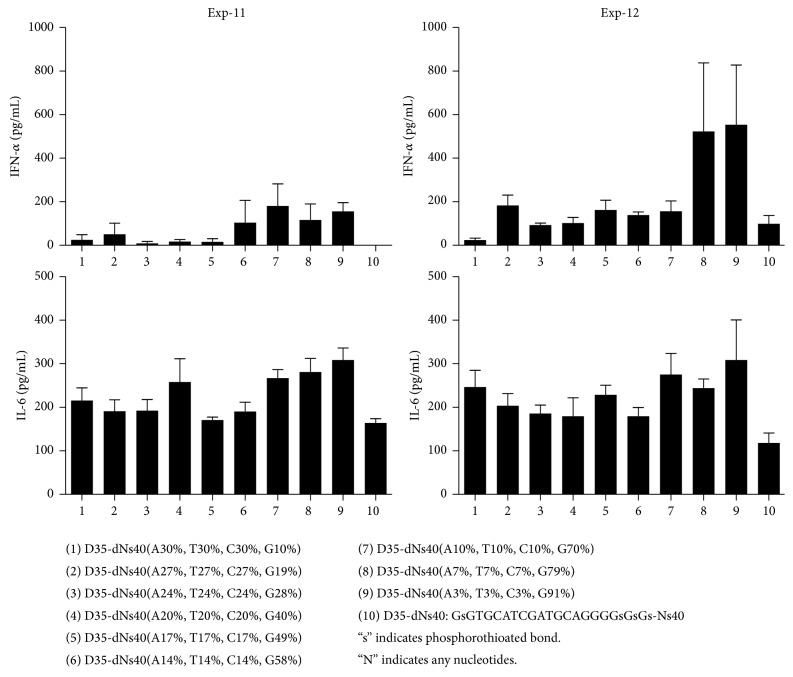
Nucleotide composition of the tail affects ODN's immunostimulatory activities. Human PBMCs were stimulated with the indicated synthetic ODNs (1 *μ*M) for 24 hours. IFN-*α* and IL-6 production in the supernatants were examined by ELISA. Of note, D35-dNs40 (number 10) did not induce substantial IFN-*α* production.

We further performed a couple of more detailed experiments to understand the requirement of the phosphorothioate polynucleotide tailing for D35 CpG ODNs, such as the length (Figure 5), the amount of phosphorothioation (Figure 6), and the nucleotides compositions (Figure 7) of the tail. First, D35core with different number of dAs was tested for the induction of IFN-*α* and IL-6 (Figure 5). No IFN-*α* induction was observed in D35core with less than dAs6; then the amount of IFN-*α* was increased by the dA length reaching dAs60. Interestingly, further prolongation of dAs-tail up to 100 did not improve but rather decreased the induction of IFN-*α*. In contrast, IL-6 production was increased and sustained up to dAs100. Second, by using D35core-dAs40, we changed the amount of phosphorothioation from 100% to 17.5% as indicated in Figure 6. Even with 50% reduction of phosphorothioation in dA40 tail abrogated the IFN-*α* inducing activities. IL-6 production was less sensitive but also rapidly decreased as the reduction of phosphorothioation amount. We did not observe any IL-6 production with less than 25% phosphorothioation. Third, the requirement of nucleotides compositions was examined by using D35-dNs40. Totally random dNs40 tailed ODNs did not induce substantial IFN-*α* production (Figure 7; sample 10). We also controlled guanosine amount in the dNs40-tail from 10% to 90%, expecting the increase of the Hoogsteen base pairing formation in the dNs40 tail. As the guanosine amount increased, the IFN-*α* production increased but D35-dNs40 (91% G) (Figure 7; sample 9) induced relatively small amount of IFN-*α*. All together these results suggested that (1) total length, (2) phosphorothioation amount, and (3) the nucleotide composition (A-polymer is better than G-rich randomer) were all important factors affecting the immunostimulating activities of phosphorothioate polynucleotide tailed ODNs. Based on these observations, we selected D35-dAs40 and D35core-dAs40 as prototypes orienting for clinical application.

### 3.9. Lyophilized D35-dAs40 and D35core-dAs40 in Vials Can Be Directly Formulated with Saline

**Figure 8:**
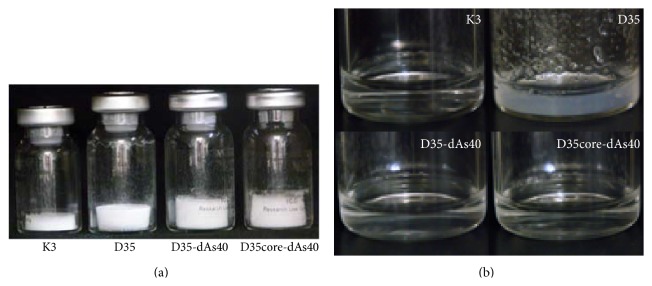
D35-dAs40 and D35core-dAs40 but not original D35 are directly solubilized in saline. (a) GMP grade lyophilized ODN vials (10 mg/vial) of K3, D35, D35-dAs40, and D35core-dAs40. White material is the lyophilized synthetic ODNs. (b) Saline (1 mL) was directly added to each vial. K3, D35-dAs40, and D35core-dAs40 were completely solubilized in saline at 5 min. D35 was not completely dissolved in saline with many visible gelatinous aggregations, and this insolubilized status was unchanged for at least 1 month.

Direct solubility of immunostimulatory ODNs in a salt-containing solution such as saline is an important requirement to accelerate their clinical applications. Therefore, we tested the direct solubility of lyophilized D35-dAs40 and D35core-dAs40 in saline. Vials containing 10 mg of lyophilized ODN (Figure 8(a)) received 1 mL of directly injected sterile saline solution. K3 and D35core-dAs40 were easily dissolved in saline (Figure 8(b)). D35-dAs40 (containing a G-hexamer sequence) was slowly but completely dissolved within 5 min (Figure 8(b)). Even when the ODN solutions were stored at 4°C for 1 month, no visible precipitation or aggregation was observed. In contrast, original D35 was not readily dissolved in saline and formed heterogeneous gelatinous aggregations (Figure 8(b)). These results demonstrated that D35-dAs40 and D35core-dAs40 could be handled more easily than original D35, especially for clinical applications.

### 3.10. Addition of dAs40 Tail to Other A/D Type ODNs Improved Their Solubility in Saline

**Figure 9:**
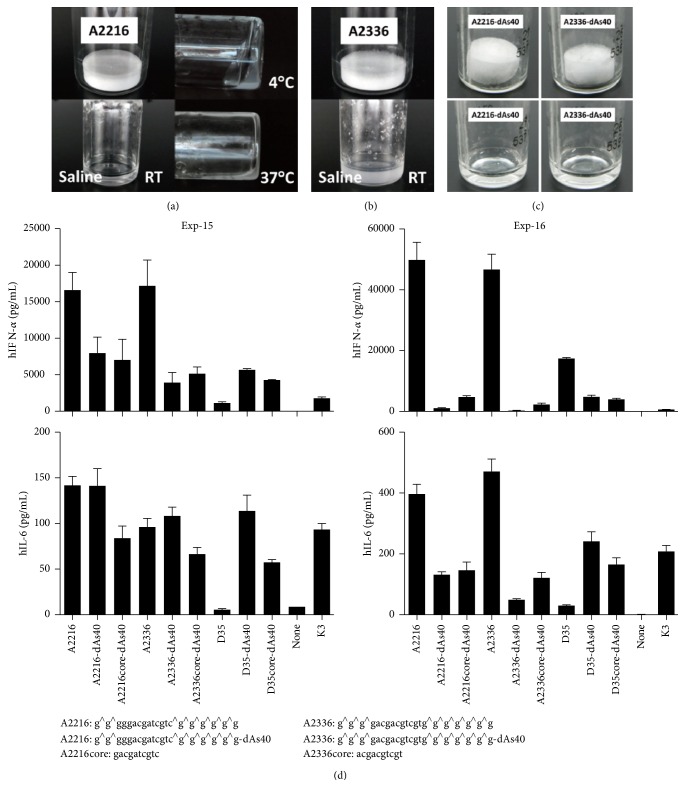
3′ addition of dAs40 sequence to A2216 and A2336 improves direct solubilization in saline. (a) Lyophilized A2216 vial (10 mg/vial) (upper left) was completely solubilized with 1 mL of saline directly at room temperature (RT) (lower left). The solution turned to a gel at 4°C (upper right) and reliquefied at 37°C (lower right). (b) Lyophilized A2336 vial (10 mg/vial) (upper) formed many visible gelatinous aggregation with 1 mL of saline at RT (lower). (c) Lyophilized A2216-dAs40 (0.5 mg/vial) and A2336-dAs40 (0.5 mg/vial) were easily and completely dissolved with 50 *μ*L of saline at RT. (d) Human PBMCs were stimulated with the indicated synthetic ODNs (1 *μ*M) for 24 hours. IFN-*α* and IL-6 production in the supernatants were examined by ELISA. Bar indicates the mean ± SEM.

We also examined the direct solubility of other A/D type ODNs such as A2216 and A2336 in saline, with and without the addition of dAs40 tail. Unexpectedly A2216 was gradually but completely dissolved in saline at room temperature. However, the solution turned into a gel at 4°C (Figure 9(a)). This gelation was reliquefied at 37°C incubation (Figure 9(a)). A2336 was not dissolved in saline and formed gelatinous aggregations, similar to D35 (Figure 9(b)). Addition of dAs40 tail to A2216 and A2336 greatly improved their solubility in saline. Both A2216-dAs40 and A2336-dAs40 readily dissolved in saline (Figure 9(c)) and did not show gelation at the 4°C. A2216-dAs40 and A2336-dAs40 also kept the IFN-*α* inducing abilities (Figure 9(d)). These results suggested that the addition of dAs40 tail is also useful modification for improving the other A/D type ODNs' manageabilities.

### 3.11. SPGylation of D35 Does Not Improve IFN-*α* Secretion from PBMCs

**Figure 10:**
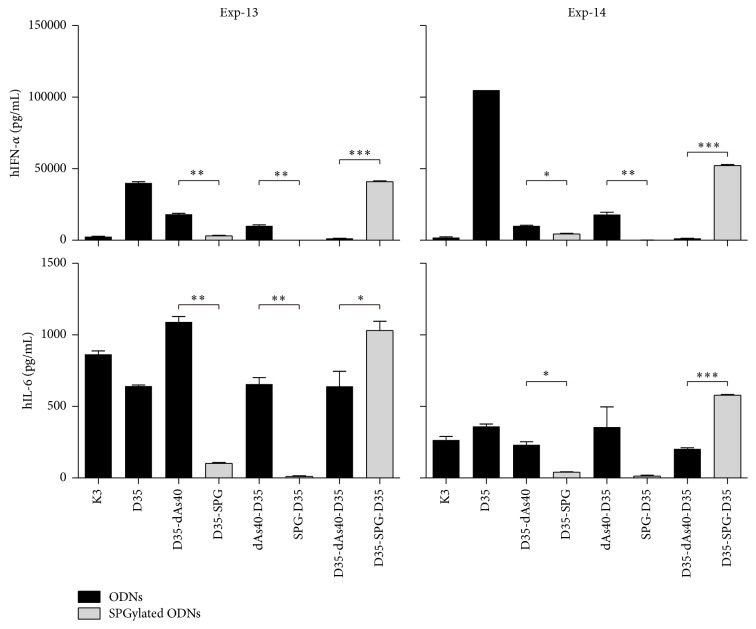
D35 modification with dAs40 at 5′ and/or 3′ ends and their SPGylation effect on cytokine production from human PBMC. Human PBMCs were stimulated with ODNs (D35-dAs40, dAs40-D35, and D35-dAs40-D35; 1 *μ*M each) or their SPGylated ODNs (D35-SPG, SPG-D35, and D35-SPG-D35; 1 *μ*M each ODN amount), and then 24 hours later, IFN-*α* and IL-6 secretion in supernatants were determined by ELISA. Bar graph shows mean ± SEM in triplicate. ^∗^ *P* < 0.05, ^∗∗^ *P* < 0.01, and ^∗∗∗^ *P* < 0.001.

**Figure 11:**
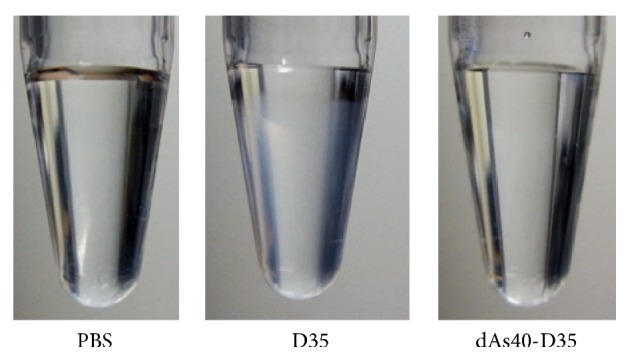
5′ addition of dAs40 sequence to D35 also prevents visible large aggregate formation in PBS. The indicated ODNs were initially dissolved in distilled water at a concentration of 10 mg/mL (all ODNs were completely solubilized with water and the solutions were clear) and further diluted with PBS at a final concentration of 1.0 mg/mL. Solutions were stored at 4°C for at least 18 hours and then images were captured. D35 developed visible white turbidity during this incubation. In contrast, dAs40-D35 did not develop visible white turbidity.

We then attempted to improve the immunostimulatory profile of D35-derived ODNs by complexing them with SPG (SPGylation; see Materials and Methods), performed similarly with previously reported K3-SPG [16]. D35-SPG, SPG-D35, and D35-SPG-D35 were made by complexing SPG with D35-dAs40, dAs40-D35, and dAs40-D35-dAs40, respectively. Complexation efficiency was evaluated by a MultiNA microchip electrophoresis system and the result was as follows: D35-SPG (99.4%), SPG-D35 (96.7%), and D35-SPG-D35 (49.8%). This indicated that ODNs in either D35-SPG or SPG-D35 solution were almost completely complexed, whereas only 50% of ODN was complexed in the D35-SPG-D35 solution. Human PBMCs were stimulated with the SPGylated ODNs, and IFN-*α* and IL-6 secretion was determined by ELISA (Figure 10). In contrast to K3-SPG [16], 5′ or 3′ SPGylation of D35 did not improve cytokine production but reduced IFN-*α* and IL-6 secretion compared with the non-SPGylated ODNs (Figure 10). Among them, D35-SPG had a greater immunostimulatory effect than SPG-D35 (Figure 10). Of note, the non-SPGylated ODNs such as D35-dAs40 and dAs40-D35 with a 5′ end addition of dAs40 and 3′ end addition of dAs40 showed comparable immunostimulatory activities (Figure 10). However, we observed slightly better cytokine production with D35-dAs40 compared to with dAs40-D35 in other experiments. Interestingly, dAs40-D35 also did not develop visible large aggregates formation in PBS (Figure 11). D35-SPG-D35 substantially enhanced IFN-*α* and IL-6 secretion from PBMCs, although the complexation efficiency was only about 50%. Although D35-SPG-D35 showed a cytokine profile improvement similar to that for K3-SPG [16], we did not pursue D35-SPG-D35 development further in this study, because of the ODN size (80 base pairs) and difficulties in achieving full complexation with SPG. Further experiments are required to understand the mechanisms and biological characteristics of these SPGylated D type CpG ODNs.

### 3.12. D35-dAs40, D35core-dAs40, and D35-SPG Are Better Vaccine Adjuvants for Influenza Split Vaccine Than K3 and Original D35 in Cynomolgus Monkeys

**Figure 12:**
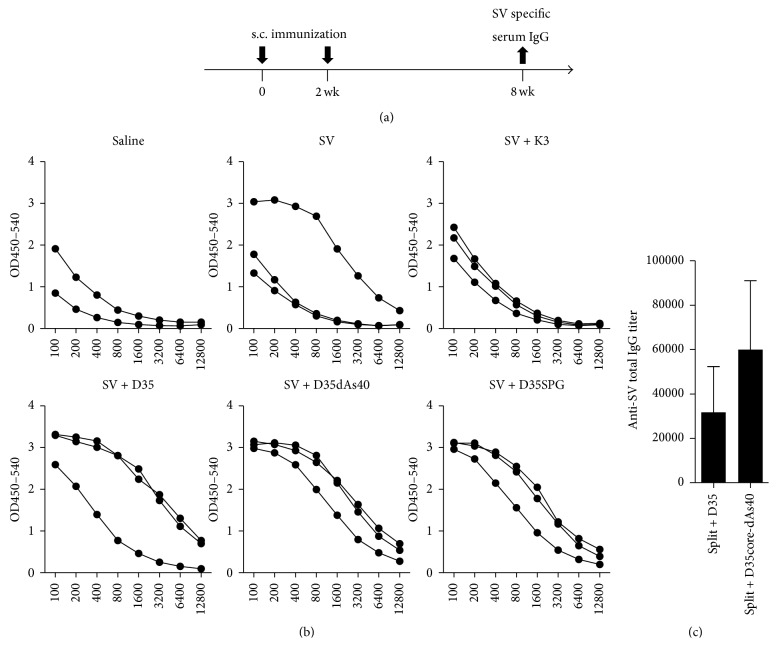
D35-dAs40, D35core-dAs40, and D35-SPG showed better adjuvanticity with influenza SV vaccine in cynomolgus monkeys. (a) Immunization and assay schedule. Six groups of monkeys (*n* = 2 or 3) were immunized with SV vaccine (A/New Caledonia/20/99, 5 *μ*g/head) s.c. in a total volume of 500 *μ*L with or without the indicated adjuvants (4.7 nmol each: K3; 30 *μ*g, D35; 30 *μ*g, D35-dAs40; 92 *μ*g, D35-SPG; 92 *μ*g as D35-dAs40 amount) twice in 2-week intervals. (b) Anti-SV total IgG in sera was determined by ELISA. *X*-axis indicates the serum dilutions. Each line indicates an individual monkey. (c) In a separate experiment, two groups of monkeys (*n* = 3) were immunized with D35 (4.7 nmol = 30 *μ*g) or D35core-dAs40 (4.7 nmol = 80 *μ*g) as in (a). Four weeks after the first immunization, anti-SV total IgG titers were determined by ELISA. Bar indicates the mean ± SEM.

**Figure 13:**
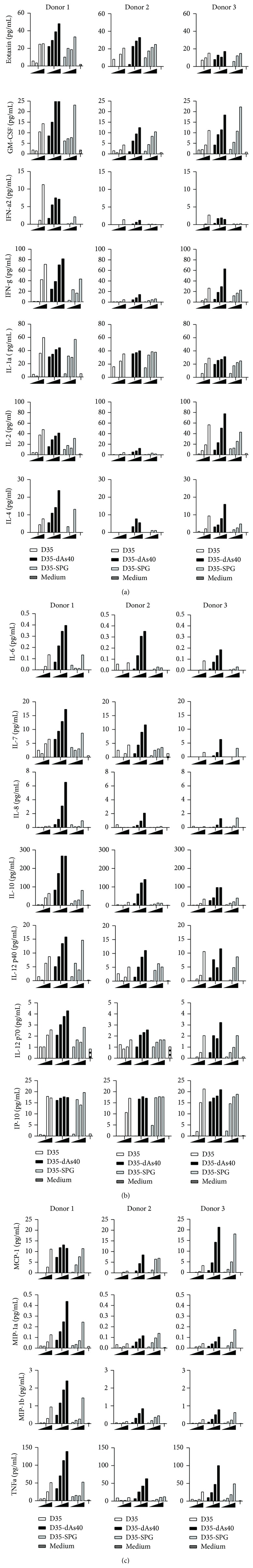
Cytokine profiles of D-type CpG ODNs in human PBMCs. PBMCs from three Japanese adult volunteers were stimulated with D35, D35-dAs40, D35-SPG, or medium. Each ODN was serially diluted (0.74, 2.2, 6.6, or 20 *μ*g/mL). After 24 hours, the cytokine concentrations in the supernatants were determined with Milliplex cytokine assay kit.

We finally tested the* in vivo* adjuvant potency of D35-dAs40, D35core-dAs40, and D35-SPG in a monkey vaccine model and compared them with K3 and original D35 (Figure 12). Six groups of monkeys (*n* = 2 or 3) were immunized subcutaneously twice (at 2-week intervals) with the indicated SV plus adjuvants, and, 8 weeks after the first immunization, SV-specific IgG responses in sera were examined by ELISA (Figure 12(a)). D35-dAs40 and D35-SPG showed better and more consistent adjuvanticity than K3 and original D35 (Figure 12(b)). We also performed another set of monkey experiments to compare original D35 and D35core-dAs40 and found that D35core-dAs40 also showed better adjuvanticity than original D35 (Figure 12(c)). These results suggested that D35-dAs40, D35core-dAs40, and D35-SPG function as comparable or better adjuvants compared with K3 and original D35* in vivo* in monkeys, at least for influenza SV vaccination. D35-SPG result in monkey also suggested that IFN-*α* and IL-6 profiles* in vitro* were not always correlated with the* in vivo* adjuvanticity. We performed more detailed cytokine profiling with a 26-cytokine multiplex using human PBMCs (instead of monkey PBMCs, owing to the limitation of obtaining sufficient amounts of monkey PBMCs for assay) stimulated with D35, D35-dAs40, or D35-SPG (Figure 13) and did not observe apparent correlation between cytokines and* in vivo* adjuvanticity among the 18 detected cytokines (Figure 13).

## 4. Discussion

In this study, we developed D35-dAs40 and D35core-dAs40, two novel prototypic nonaggregating immunostimulatory A/D type ODNs for clinical use in humans. These ODNs showed similar cytokine profiles to the original D35 with high IFN-*α* and low IL-6 induction profile from human PBMCs, although the overall balance between these cytokines was slightly shifted toward that of B/K type ODNs (slightly reduced IFN-*α* and increased IL-6 compared with original D35) (Figure 1). The most important feature of D35-dAs40 and D35core-dAs40 was their excellent solubility in saline. Lyophilized D35-dAs40 and D35core-dAs40 stored in vials can be directly solubilized by injecting saline solution (Figure 8), a requirement for clinical administration, and this feature greatly broadens their application. In addition, both D35-dAs40 and D35core-dAs40 showed better adjuvanticity than original D35 in cynomolgus monkeys when used as an influenza SV adjuvant (Figure 12). This result was not expected, because D35-dAs40 and D35core-dAs40 generally showed relatively reduced IFN-*α* and more IL-6 compared with original D35* in vitro* (Figure 4). Even compared with K3, considered to induce better antibody responses owing to the direct activation of B cells and strong IL-6 cytokine induction, D35-dAs40 showed better and more reliable anti-influenza antibody responses in monkeys (Figure 12). This suggested that both D35-dAs40 and D35core-dAs40 are excellent vaccine adjuvants* in vivo*. We also tested D35-SPG (consisting of D35-dAs40 and SPG) in monkeys. Although D35-SPG showed reduced IFN-*α* and IL-6 levels* in vitro*, D35-SPG had comparable adjuvanticity to D35-dAs40* in vivo* (Figure 12). These pieces of data indicated that the cytokine amount and quality induced by ODNs* in vitro* do not necessarily correlate with their adjuvanticity* in vivo*. The difference in biodistribution might be another important factor that affects the adjuvanticity of the modified ODNs. In the case of K3-SPG, we observed different biodistribution in the draining LN between K3-SPG and K3 [16]. Further investigation is required in order to understand the relationship between* in vivo* adjuvanticity and the ODN biodistribution.

By dose-response analysis, D35-dAs40 and D35core-dAs40 showed dose-proportional IFN-*α* and IL-6 responses similar to original D35 (Figure 4), and this is one reason why we consider D35-dAs40 and D35core-dAs40 as “D type” and not C or P type CpG ODNs. C and P type ODNs also induce IFN-*α* production; however, their “ODN dose-cytokine responses” are usually different from the D type ODN pattern. C and P type ODNs showed reduced IFN-*α* secretion when a larger amount of ODNs was used for stimulation, and this type of IFN-*α* response was also observed by us for K3-SPG, which originated from K3 CpG ODN, although it could still induce robust IFN-*α* production [16]. Because the backbones of C, P, and K3-SPG are all phosphorothioate, this inversely proportional IFN-*α* response might be caused by the phosphorothioate backbone structures; however, the underlying mechanisms are currently unknown and need to be investigated in the future. Taken together, the present study demonstrated that D35-dAs40 and D35core-dAs40 are promising prototypic D type CpG ODNs with high solubility in saline and high adjuvanticity* in vivo*.

A G-hexamer sequence and the resulting aggregation of ODNs are not necessary for A/D type ODNs to induce IFN-*α* production from human PBMCs, which was previously believed to be an obligatory requirement. Earlier studies concluded that aggregation was necessary for high IFN-*α* production by A/D type CpG ODNs [12–14]. However, our data demonstrate that aggregation is not an absolute requirement for high IFN-*α* production by A/D type CpG ODNs (Figure 1), suggesting that the G-hexamer sequence itself is not directly involved in TLR9-mediated CpG ODN recognition. D35 ODNs not containing G-hexamers (such as D35A, D35T, and D35C) with DOTAP induced strong IFN-*α* production (Figure 2), thus further supporting this hypothesis, where DOTAP compensated for the aggregation-dependent uptake processes of D-type ODNs. In contrast, IFN-*α* production was completely dependent on the presence of CpG motifs (Figure 1). These data suggested that the overall immunostimulatory activities of A/D type ODNs could be regulated by two nonoverlapping mechanisms: (1) ODN uptake by cells and (2) CpG motif recognition by TLR9 in the endosome. Taken together, we concluded that the CpG-containing 12-mer palindromic sequence and the phosphorothioated poly-A tail were the two minimal and sufficient components of our newly developed nonaggregating D type ODNs, such as D35-dAs40 and D35core-dAs40.

The presence of a G-hexamer has been reported to contribute to the efficient uptake of phosphodiester backboned ODNs by cells, possibly through scavenger receptors [19, 23, 26]. It was demonstrated that phosphodiester backboned ODNs require a G-hexamer or related sequences that form aggregations for ODN uptake by cells and the subsequent immunostimulatory functions. However, phosphorothioated single strand CpG ODNs (such as B/K type ODNs) were reported to use DEC-205 for their uptake by cells [27]. In addition, many molecules such as HMGB1, granulin, and LL37 mediate or enhance ODN or DNA uptake and delivery to TLR9 [28]. D35-dAs40 and D35core-dAs40 do not form aggregates but maintain their immunostimulatory activity dependent on the presence of phosphorothioate but not the phosphodiester poly-A tail, suggesting that uptake of D35-dAs40 and D35core-dAs40 is likely mediated by phosphorothioate ODN uptake mechanisms such as DEC-205 and/or possibly other undiscovered accessory molecules. The determination of the precise uptake mechanisms should be addressed in the future.

G-hexamer-mediated aggregation or high order structure formation may also affect subcellular localization and facilitate preferential early endosome localization of ODNs with multimeric form [29]; however, the precise regulation mechanism of this preferential early endosome sorting remains to be determined. It was suggested that IFN-*α* production signaling starts at CpG/TLR9 interactions residing in the early endosomes [25, 29]. Forced targeting of B/K type ODNs (not a good inducer of IFN-*α*) to early endosomes with DOTAP also induced IFN-*α* [25], suggesting the requirement of a backbone chemical feature and high order structures are not strict when CpG ODNs are targeted to the proper compartment for IFN-*α* production. Similarly, a recent report examined two different higher-order-structured CpG ODNs attached to a nanoparticle surface and found that multimerized-ODNs/nanoparticles induced IFN-*α* responses while monomeric-ODNs/nanoparticles induced IL-6 responses [30]. These reports suggested that both ODN structures and targeted cellular compartments are important for determining the final preferential cytokine responses, either IFN-*α* or IL-6. Recently reported AP-3 added another layer to the regulation of IFN-*α* responses of pDCs [31]. TLR9 localization to lysosome-related organelles that might be derived from late endosomes was required for IFN-*α* production from pDCs [31], suggesting that IFN-*α* production by TLR9 signaling was regulated by a more complicated intracellular sorting mechanism than previously thought [32]. D35-dAs40 and D35core-dAs40 are composed of nonaggregated D type sequences and phosphorothioate poly-A tail and can induce both IFN-*α* and IL-6, which may resemble the intracellular distribution of C type ODNs, although their dose-response reflected D type ODNs. Further study is required to clarify the exact cellular compartment and molecular requirements of D35-dAs40 and D35core-dAs40 induction of IFN-*α* production.

### 4.1. Individual Response Differences

Heterogeneity of cytokine responses to B/K type CpG ODNs in human PBMC has been reported [33]. Although overall responses were largely consistent among different human PBMC samples, we also noticed variable responses against nonaggregated derivatives of A/D type ODNs examined in this study. Future investigation of the causes of these individual differences is critical for the clinical application of this type of ODN. However, we believe that nonaggregated forms of A/D type ODNs including D35-dAs40 and D35core-dAs40 are promising starting materials for the further development of clinically applicable A/D type ODNs.

## 5. Conclusions

In this study, we developed nonaggregating A/D type ODNs and demonstrated that D35-dAs40 and D35core-dAs40 are two promising prototypes of nonaggregating A/D type ODN with advantages of ease of drug preparation for clinical applications as vaccine adjuvants or IFN-*α* inducing immunomodifiers.

## Figures and Tables

**Table 1:** Modified D35 ODNs developed in this study.

D35(CG)G-dAs40^∗^	GsGTGCATCGATGCAGGGGsGsGs-As40	Figure 1(a)	1
D35(GC)G-dAs40	GsGTGCATGCATGCAGGGGsGsGs-As40		2
D35(TG)G-dAs40	GsGTGCATTGATGCAGGGGsGsGs-As40		3
D35(CT)G-dAs40	GsGTGCATCTATGCAGGGGsGsGs-As40		4
D35(CG)A-dAs40	GsGTGCATCGATGCAAAAAsAsAs-As40		5
D35(GC)A-dAs40	GsGTGCATGCATGCAAAAAsAsAs-As40		6
D35(TG)A-dAs40	GsGTGCATTGATGCAAAAAsAsAs-As40		7
D35(CT)A-dAs40	GsGTGCATCTATGCAAAAAsAsAs-As40		8
D35(CG)T-dAs40^∗∗^	GsGTGCATCGATGCATTTTsTsTs-As40		9
D35(GC)T-dAs40	GsGTGCATGCATGCATTTTsTsTs-As40		10
D35(TG)T-dAs40	GsGTGCATTGATGCATTTTsTsTs-As40		11
D35(CT)T-dAs40	GsGTGCATCTATGCATTTTsTsTs-As40		12
D35(CG)C-dAs40	GsGTGCATCGATGCACCCCsCsCs-As40		13
D35(GC)C-dAs40	GsGTGCATGCATGCACCCCsCsCs-As40		14
D35(TG)C-dAs40	GsGTGCATTGATGCACCCCsCsCs-As40		15
D35(CT)C-dAs40	GsGTGCATCTATGCACCCCsCsCs-As40		16

D35T-dAs40^∗∗^	GsGTGCATCGATGCATTTTsTsTs-As40	Figure 1(b)	1
D35T-dA40	GsGTGCATCGATGCATTTTsTsTs-A40		2
D35T-dTs40	GsGTGCATCGATGCATTTTsTsTs-Ts40		3
D35T-dT40	GsGTGCATCGATGCATTTTsTsTs-T40		4
D35T-dCs40	GsGTGCATCGATGCATTTTsTsTs-Cs40		5
D35T-dC40	GsGTGCATCGATGCATTTTsTsTs-C40		6

D35-dAs40^∗^	GsGTGCATCGATGCAGGGGsGsGs-As40	Figures 3(a), 3(b), and 3(c) Figures 4(a) and 4(b)	
D35T-dAs40^∗∗^	GsGTGCATCGATGCATTTTsTsTs-As40		
D35core	TGCATCGATGCA		
D35core-dAs40	TGCATCGATGCA-As40		
D35coreT-dAs40	TGCATCGATGCATTTTsTsTs-As40		

D35core-dAs10	TGCATCGATGCA-As10	Figures 4(c) and 4(d)	
D35core-dAs20	TGCATCGATGCA-As20		
D35core-dAs40	TGCATCGATGCA-As40		

“s” indicates phosphorothioate backbone.

^∗^D35(CG)G-dAs40 and D35-dAs40 have the same sequence.

^∗∗^D35(CG)T-dAs40 and D35T-As40 have the same sequence.

**Table 2:** DLS measurement of each ODN in Figure 3.

	Diameter (nm)	% Pd	Mw-R (kDa)	% intensity	% mass
K3	2.7	26.0	7	100.0	100.0
D35	213.2	74.2	187014	77.1	79.7
D35-dAs40	11.8	48.7	212	97.2	99.9
D35T-dAs40	7.6	43.3	76	80.6	99.6
D35core-dAs40	6.9	23.5	61	96.6	100.0
D35coreT-dAs40	7.3	35.4	70	79.4	99.6

The value of the main peak in each ODN measurement is shown. Pd: polydispersity.
